# Exosomes Derived From Mesenchymal Stem Cells: Novel Effects in the Treatment of Ischemic Stroke

**DOI:** 10.3389/fnins.2022.899887

**Published:** 2022-05-02

**Authors:** Yu Xiong, Jianping Song, Xinyue Huang, Zhigang Pan, Roland Goldbrunner, Lampis Stavrinou, Shu Lin, Weipeng Hu, Feng Zheng, Pantelis Stavrinou

**Affiliations:** ^1^Department of Neurosurgery, The Second Affiliated Hospital, Fujian Medical University, Quanzhou, China; ^2^Department of Neurosurgery, Shanghai Medical College, Huashan Hospital, Fudan University, Shanghai, China; ^3^National Center for Neurological Disorders, Shanghai, China; ^4^Neurosurgical Institute of Fudan University, Shanghai, China; ^5^Shanghai Clinical Medical Center of Neurosurgery, Shanghai, China; ^6^State Key Laboratory of Medical Neurobiology, MOE Frontiers Center for Brain Science, Institutes of Brain Science, Fudan University, Shanghai, China; ^7^Department of Neurosurgery, National Regional Medical Center, Fudan University Huashan Hospital Fujian Campus, The First Affiliated Hospital Binhai Campus, Fujian Medical University, Fuzhou, China; ^8^Department of Neurosurgery, Faculty of Medicine and University Hospital, Center for Neurosurgery, University of Cologne, Cologne, Germany; ^9^2nd Department of Neurosurgery, Athens Medical School, “Attikon” University Hospital, National and Kapodistrian University, Athens, Greece; ^10^Centre of Neurological and Metabolic Research, The Second Affiliated Hospital of Fujian Medical University, Quanzhou, China; ^11^Diabetes and Metabolism Division, Garvan Institute of Medical Research, Sydney, NSW, Australia; ^12^Department of Neurosurgery, Metropolitan Hospital, Athens, Greece

**Keywords:** exosomes, ischemic stroke, miRNAs, mesenchymal stem cells, treatment

## Abstract

Ischemic stroke is defined as an infarction in the brain, caused by impaired cerebral blood supply, leading to local brain tissue ischemia, hypoxic necrosis, and corresponding neurological deficits. At present, revascularization strategies in patients with acute ischemic stroke include intravenous thrombolysis and mechanical endovascular treatment. However, due to the short treatment time window (<4.5 h) and method restrictions, clinical research is focused on new methods to treat ischemic stroke. Exosomes are nano-sized biovesicles produced in the endosomal compartment of most eukaryotic cells, containing DNA, complex RNA, and protein (30–150 nm). They are released into surrounding extracellular fluid upon fusion between multivesicular bodies and the plasma membrane. Exosomes have the characteristics of low immunogenicity, good innate stability, high transmission efficiency, and the ability to cross the blood–brain barrier, making them potential therapeutic modalities for the treatment of ischemic stroke. The seed sequence of miRNA secreted by exosomes is base-paired with complementary mRNA to improve the microenvironment of ischemic tissue, thereby regulating downstream signal transduction activities. With exosome research still in the theoretical and experimental stages, this review aims to shed light on the potential of exosomes derived from mesenchymal stem cells in the treatment of ischemic stroke.

## Introduction

Ischemic stroke (IS) is characterized by the sudden loss of blood circulation to an area of the brain, resulting in a corresponding loss of neurologic function. Acute IS is caused by thrombotic or embolic occlusion of a cerebral artery and is the leading cause of death and acquired disability worldwide (Ai et al., 2021). Cerebral artery thrombosis accounts for > 80% of all strokes (Liu et al., 2021). Cerebral ischemia can lead to various secondary harmful effects, including reperfusion injury, ischemia hypoxia, increased intracellular calcium levels, blood–brain barrier (BBB) damage, glial cell activation, oxidative stress, ion imbalance, apoptosis, and inflammation, ultimately causing neuronal damage and death. These pathological reactions eventually lead to corresponding neurologic symptoms, such as cognitive, motor, and sensory dysfunction, which seriously affect the physical health and quality of life of patients with IS. The 30-day mortality rate is 5–15%, and the disability rate exceeds 50% (Strain et al., 2021). Without effective treatment within 4.5 h after onset, the mortality and disability rates of IS are even higher (Liu et al., 2021). Rapid reperfusion with intravenous thrombolysis and endovascular thrombectomy represent the mainstays of treatment. However, due to the strict inclusion criteria and the many contraindications for thrombolysis, including the risk of hemorrhagic transformation, the clinical applications of endovascular therapy remain limited (Ai et al., 2021). Therefore, developing new treatment methods is necessary.

Exosomes are membrane vesicles secreted by any cell type and are generally released into the intercellular space after binding to the plasma membrane (Cai et al., 2021). They contain several biologically active genomic and non-genomic molecules, including DNA, miRNAs, mRNA, lncRNA, various proteins, lipids, and enzymes, which can change the biological behavior of cells through paracrine or autocrine mechanisms (Baruah and Wary, 2019). The special phospholipid bilayer of the exosomal membrane can prevent the decomposition of biologically active substances under harsh conditions and facilitate the storage of these exosomes *in vitro* (Quaglia et al., 2022). These biological properties provide the basis for potentially new treatment modalities. In recent years, many studies have focused on the different functions of exosomes in the treatment of various diseases. Exosomes have been reported to promote neurological recovery and angiogenesis after IS (Xin et al., 2013, 2021; Yang et al., 2017; Baruah and Wary, 2019). Exosomes derived from mesenchymal stem cells (MSCs) are considered to have the greatest potential in the treatment of stroke; the secretion of exosomes by MSCs is considered a major mechanism by which the latter promotes neurovascular remodeling and the recovery of neurological function after stroke (Hong et al., 2019).

Here, we review studies on exosomes derived from MSCs, which demonstrate that in various ischemic diseases, exosomes released by MSCs affect most damaged tissues (Anderson et al., 2016). Among them, bone marrow-derived MSCs send signals to endogenous cell groups, including immune cells and endothelial cells, to trigger their tissue healing ability (Shang et al., 2022). Animal experiments have shown that transplantation of bone-marrow MSCs (BMSCs) can significantly improve the neurological deficit and quality of life in the middle cerebral artery occlusion (MACO) rat model (Zhang Z. et al., 2021), and this effect may likely be due to exosomes secreted by stem cells (Chen H. et al., 2022). Whether other types of MSCs have the ability to improve the microenvironment, reduce inflammatory response, and promote microvascular response in IS is still unclear.

## Ischemic Stroke

IS is a cascade of damage responses secondary to neurovascular injury during cerebral cell ischemia and hypoxia, mainly caused by cerebral artery occlusion of thrombotic or embolic origin.

### Pathophysiology

#### Reperfusion Injury

Reperfusion is the action by which blood supply is restored to the brain (Barzegar et al., 2021). Reperfusion of the ischemic area is the most effective method to protect brain structure and function, though it can also cause reperfusion injury (Castelli et al., 2021). Reperfusion injury after IS, defined as the rapid increase in tissue damage after the blood supply to ischemic brain tissue cells is restored, may occur after thrombolysis with tissue plasminogen activator (t-PA), after mechanical thrombectomy, or as a result of spontaneous reperfusion (Lin et al., 2016). Reperfusion, although theoretically beneficial to hypoxic tissues, can produce paradoxical tissue responses such as calcium overload, promotion of reactive oxygen species (ROS), deposition of pro-inflammatory immune cells in ischemic tissue, activation of glial cells, and damage of the blood–brain barrier (Kalogeris et al., 2016).

#### Elevated Intracellular Calcium Levels

**FIGURE 1:**
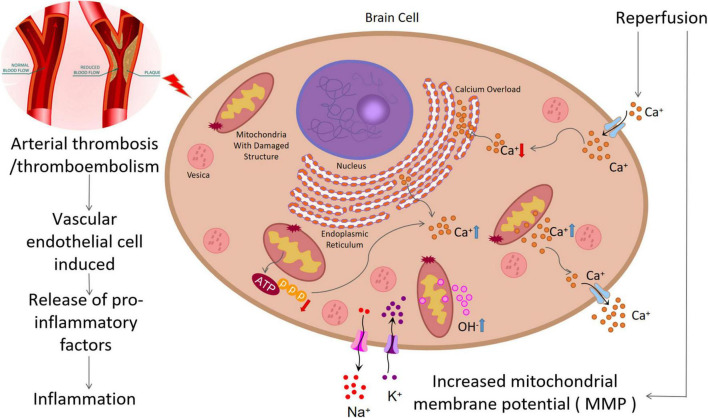
Pathological process of ischemic stroke: (1) Arterial thrombosis or arterial embolism will lead to ischemia and hypoxia of nerve cells, leading to damage of mitochondrial structure and function, decrease of ATP generation, increase of intracellular calcium overload and free radical generation, and aggravation of ischemia and hypoxia. (2) In addition, this process can damage endothelial cells and increase inflammatory response. (3) Mitochondrial structure damage can increase the concentration of OH^–^, causing oxidative stress. (4) Reperfusion injury can increase mitochondrial membrane potential (MMP), resulting in increased OH^–^ production and increased oxidative stress.

When an IS occurs, brain tissue ischemia and hypoxia will interfere with the oxidative phosphorylation of mitochondria, leading to disorders of energy metabolism and reduced ATP production. Tissue ATP is rapidly depleted, and calcium ions (Ca^2+^) are released from the mitochondria and endoplasmic reticulum (Shi et al., 2021). If the depleted ATP pump function does not recover after reperfusion, significant extracellular fluid Ca^2+^ will flow into the intracellular fluid. This process not only produces free radicals but also makes large blood vessels contract, thereby aggravating ischemia and hypoxia and leading to the formation of a vicious circle. This is the main pathogenesis of extensive tissue cell injury (Liu et al., 2018) (Figure 1).

#### Generation of Reactive Oxygen Species

After ischemia and reperfusion, the function and structure of the mitochondria are impaired, leading to an increase of the oxygen atom tetravalent, leakage, and the generation of hydroxyl free radicals. Another important source of ROS is reduced nicotinamide adenine dinucleotide phosphate oxidase (NOX). Studies have revealed that ischemia–reperfusion can increase the mitochondrial membrane potential, leading to excessive production of ROS (Lin et al., 2016). When ROS exceeds the antioxidant capacity, oxidative stress will occur, which directly damages all cellular components, including protein, DNA, RNA, and lipids.

#### Deposition of Pro-inflammatory Cells

After endothelial cell (EC) injury, leukocyte infiltration and the subsequent inflammatory response play a crucial role. Activated leukocytes adhere to the EC, infiltrate brain tissue, destroy the BBB, and release pro-inflammatory factors (TNF-α, IL-1, IL-6, MCP1) and inflammatory mediators (ROS, NOS), leading to the deterioration of the ischemic penumbra (Cai et al., 2021). IL-1 can induce the release of arachidonic acid, promote the production of TNF-α and IL-1β, lead to the deposition of inflammatory cells, and aggravate tissue damage (Zhang et al., 2018).

#### Glial Cell Activation

**FIGURE 2:**
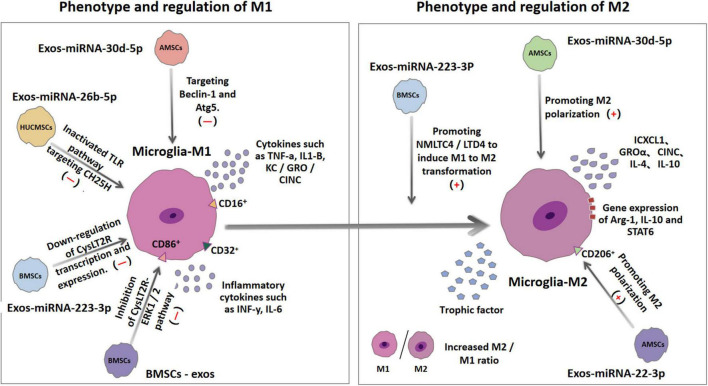
Ischemia and hypoxia can lead to the polarization of microglia into M1 microglia and M2 microglia. The surface antigens of the two microglias are different. M1 microglia expresses CD16, CD32, and CD86, while M2 microglia expresses CD206. MiRNA in exosomes derived from different types of mesenchymal stem cells can regulate some inflammatory pathways or mechanisms to up-regulate or down-regulate the production of M1 and M2, thereby changing the ratio of M1/M2 in the brain and reducing the inflammatory response and secondary tissue damage involved in M1.

Microglia are the main innate immune cells in the central nervous system; they play an important role in regulating neuroinflammation (Heneka et al., 2014). Both IS and reperfusion injury can increase the morphological changes and activity of the microglia (Surinkaew et al., 2018). Once cerebral ischemia/reperfusion (I/R) injury occurs, the resting microglia enter the active state (Hu et al., 2012). The activated microglia will be recruited to the ischemic and penumbra areas to play either neuroprotective or inflammatory roles (Xiong et al., 2016). Brain damage caused by stroke activates and differentiates microglia into M1 or M2 phenotypes (Song et al., 2019). M1 phenotypes have been confirmed to express CD86^+^, CD206^–^, and CD16/32^+^ and produce high levels of pro-inflammatory cytokines, such as INF-γ, IL-6, TNF-α, IL1-β, and KC/GRO/cytokine-induced neutrophil chemoattractant (CINC) (Spellicy and Stice, 2021). The differentiation of microglia strengthens the deterioration of the local inflammatory environment, promotes the progress of acute brain injury, and hinders nerve regeneration after injury and subsequent long-term functional recovery (Zhang Z. et al., 2021). In contrast to the M1 type, the M2 type is the “good” phenotype, which can protect the nerves from ischemia and hypoxia, and promote the long-term recovery after a stroke (Song et al., 2019). Studies have revealed that the M2 phenotype (CD86^–^, CD206^+^) increased the gene expression of Arg-1, IL-10, STAT6; released CXCL1, GROα, neutrophil activating protein alpha, CINC; and induced a more restorative local environment (Spellicy and Stice, 2021). Based on their ability to produce IL-4 and IL-10, adjacent cells can be protected by removing cell debris and releasing nutrients Moreover, M2 microglia can also perform necroptosis and produce many protective and nutritional factors that promote neurogenesis (Zhang Z. et al., 2021; Figure 2).

### Shortcomings of Existing Treatment

The key points of IS treatment are recanalization, saving ischemic penumbra, and reducing infarct size. The vascular reconstruction strategies for acute IS include intravenous thrombolysis and mechanical recanalization (Minnerup et al., 2016). Although these two treatment methods have excellent results in specific patient groups, they have strict selection criteria, which are related mostly but not exclusively to the narrow therapeutic time-window, thus limiting their clinical application (Chen et al., 2016). The efficacy of intravenous thrombolysis treating stroke is highly dependent on the treatment time (Ai et al., 2021). Initially, rt-PA was proven to be safe within 3 h after stroke; however, recent studies have extended this time window to 4.5 h (Bsat et al., 2021). Most patients present outside this time-window and are not eligible for thrombolysis. Moreover, due to the low recanalization rate, especially in treating severe stroke caused by occlusion of large intracranial vessels, the efficacy of drug thrombolysis is limited (Minnerup et al., 2016). Adjuvant drugs such as anticoagulants, antiplatelet agents, and anti-inflammatory drugs may have a certain role in extending the therapeutic window (Mahjoubin-Tehran et al., 2021). A new (“Lean Principles”) approach for optimizing the treatment process has also been reported, that allows patients with stroke to receive thrombolytic therapy within 60 min and improves their survival rate (Souza et al., 2021). Nevertheless, this approach does not directly address the problem of vascular stenosis caused by thromboembolism or atherosclerotic plaque. Thrombectomy is only suitable for patients with large vessel occlusion (Minnerup et al., 2016). Experiments suggest that mechanical thrombectomy combined with intravenous thrombolysis can significantly improve the neurological prognosis after 3 months and reduce mortality (fewer patients met inclusion criteria for both mechanical thrombectomy and intravenous thrombolysis) (Minnerup et al., 2016). Additionally, clinical randomized controlled trials have revealed that in patients with acute IS from large-vessel occlusion, endovascular thrombectomy alone was non-inferior in terms of functional outcomes, within a 20% margin of confidence, to endovascular thrombectomy preceded by intravenous alteplase administered within 4.5 h after symptom onset (Yang et al., 2020). Therefore, the majority of patients with IS still lack a treatment option that is both effective and safe, highlighting the need for an alternative treatment approach.

## Exosomes and Mesenchymal Stem Cells

### Background

Increasing amount of data indicate that stem cell therapy is a promising option for many diseases that cannot be effectively treated by existing treatment strategies. Recently, stem cell therapy has demonstrated potential as a treatment option for neurological diseases such as Parkinson’s, amyotrophic lateral sclerosis, and Huntington’s disease (Chen et al., 2014, 2020; Minnerup et al., 2016; Tang et al., 2022). In addition to replace lost cells during ischemia–reperfusion (the replacement mechanism) (Hurd et al., 2021), stem cells also secrete various cytokines and growth factors to induce diverse beneficial effects, such as anti-inflammatory nerve cell protection and induction of the endogenic recovery system (Yoo et al., 2013), which can provide a good environment for recovery (paracrine mechanism), which is taking the lead (Salikhova et al., 2021). Exosomes are extracellular vesicles that are released by cells after the fusion of polycystic body and plasma membrane (Mahjoubin-Tehran et al., 2021). They are 50–100 nm in diameter and can be secreted by various types of cells, including bone marrow-derived mesenchymal stem cells (BMSCs), adipose-derived mesenchymal stem cells (ADMSCs), and human umbilical cord-derived mesenchymal stem cells (HUCMSCs) (Wang et al., 2018). Although different cells release exosomes with different molecules, exosomes contain traditional markers that make them significantly different from other extracellular vesicles (Hassanpour et al., 2020; Rezaie et al., 2021). CD9, CD63, and CD81 are the most commonly used markers of exosomes. As a conservative family of tetraspanin proteins, CD9, CD63, and CD81 are also associated with many different functions, including cell adhesion, motility, and proliferation, and are involved in exosome cargo selection and trafficking. They are often used as markers for identification, quantification, or purification of exosomes (Garcia-Martin et al., 2022). Several miRNAs are loaded in the exosomes of different cell-types, and they play an important role in the recovery of damaged neurons (Deng et al., 2019). MSCs are the most effective at producing exosomes (Cosenza et al., 2018). MSCs have the ability to self-renew, regenerate, and differentiate in damaged tissues; regulate inflammation; and activate microglia (Castro-Manrreza and Montesinos, 2015; Li et al., 2015). Moreover, some of the benefits of MSCs include their relative abundance (in human’s bone marrow), avoidance of ethical problems (not from fetuses such as neural stem cells and embryonic stem cells), and a lack of tumorigenicity compared to induced pluripotent stem cells (Stonesifer et al., 2017). This allows MSCs to provide the basis for effective new therapies for various human diseases. Although information on the exact mechanism of MSC-mediated restorative effect is limited, with < 1% of transplanted MSCs reaching and colonizing the target site (most of them are embedded in the pulmonary capillary bed due to their large size), the therapeutic effects observed may be mainly attributed to the production of paracrine factors from transplanted MSC-derived exosomes (Chen H. et al., 2022). Since the potency of exosomes in tissue regeneration and repair is greater than those of stem cells themselves, stem-cell derived exosomes have become the focus of recent studies (Kalani and Tyagi, 2015).

Exosomes derived from MSCs can pass through the BBB and can also regulate the gene expression of recipient cells by delivering functional substances. Because of their small size, exosomes can escape the phagocytosis of macrophages. Compared with polymer nanoparticles and liposomes, they can escape naturally and stably from lysosome degradation. Generally speaking, because exosomes are not immunogenic, they are good candidates for gene drug delivery (Yang et al., 2017). They can also mediate intercellular communication. Recent studies have confirmed that exosomes can affect angiogenesis and glial involvement by regulating the production of nucleotides and proteins, and participate in the remodeling process after cerebral infarction (Luo et al., 2019; Kim et al., 2020). It has been reported that > 20% of miRNAs in human body changes (increases or decreases) after IS, indicating that miRNA is an important mediator of IS (Mahjoubin-Tehran et al., 2021). MiRNAs are small, highly conserved, 20–25 nt non-coding RNAs in eukaryotes (Kabekkodu et al., 2018; Huuskonen et al., 2022). They target approximately 60% of mammalian genes after transcription and participate in most biological and pathophysiological processes (Xin et al., 2014). By pairing with the 3′UTR of target mRNA molecules, miRNA regulates the expression of target genes and downstream signaling pathways at the post-transcriptional level, resulting in degradation or inhibition of target mRNA translation (Soifer et al., 2007; Baruah and Wary, 2019).

### Treatment of Different Diseases With Exosomes

Ischemic injury in other organs reportedly can be improved by using exosomes derived from MSCs (Table 1). For example, Mathew et al. (2019) discovered that MSC-extracellular vesicles (MSC-EV) can be used to treat retinal I/R injury. Experimental data support that MSC-EV significantly enhances functional recovery, and reduces neuroinflammation and apoptosis. Yan et al. (2020) discovered that in the exosomes derived from HUCMSCs, the mir-421/circhipk3/foxo3a pathway can be used to prevent pyrolysis and repair ischemic muscle damage. Similarly, Zhang et al. (2019) have reported experimental data supporting fracture repair and angiogenesis potential through hypoxia-inducible factor-1α (HIF1α). Hu et al. (2018) have reported that exosomes rich in miR-21-3p secreted by human umbilical cord blood cells can accelerate wound healing and promote angiogenesis. In recent years, the application of exosomes in the treatment of cardiovascular diseases has been commonly reported. Research has increasingly focused on myocardial ischemia injury, which is directly linked to the increase of the elderly population and subsequently to the increase of cardiovascular diseases. For instance, Wei et al. (2019) discovered that overexpression of miRNA-181a in MSC-derived exosomes can inhibit the inflammatory response after myocardial I/R injury. Khan et al. (2015) and Sun et al. (2020) have revealed that miR-221-3p and miR-294 from MSCs could promote the repair of damaged cardiomyocytes. Shi et al. (2018) identified that miR-21, an exosome derived from MSCs, down-regulated the expression of PTEN by activating the PI3K/AKT signaling pathway, thereby protecting cardiomyocytes from death induced by ischemia and oxidative stress. In addition, the latest study found that miRNA-21 can also improve myocardial remodeling after myocardial ischemia-reperfusion injury by reprogramming macrophages (Tan et al., 2021). Reprogrammed macrophages coordinate the remodeling of the myocardial microenvironment in the anti-inflammatory treatment of ischemia reperfusion (IR) injury (Tan et al., 2021). MiR-30c-5p can down-regulate BCL2-like 11 (BCL2L11) to improve myocardial injury, histopathological changes and apoptosis in rat I/R model (Meng et al., 2021). Meanwhile, MiR-29a-3p can attenuate ischemia reperfusion by up-regulating nuclear factor erythroid-2 related factor 2 (NRF2) and inhibiting Cyclin T2 (CCNT2) (Tian et al., 2021). MSCs/Exosomes/miRNAs are widely used in cardiac ischemic diseases, which may provide new guidance for the treatment of ischemic stroke. Xu F. et al. (2019) first demonstrated that miR-423-5p derived from ADMSCs is a key active molecule in exosome-induced angiogenesis. Additionally, in recent years, miR-423-5p has also been intensively researched in the diagnosis and treatment setting of various tumors (Shan et al., 2020; Jiang et al., 2021; Xue et al., 2022). Additionally, various studies have focused on exosome intercellular communication, storage of biological information, and the use of biomarkers, especially in the potential applications of regeneration and nerve protection (Kalani and Tyagi, 2015; Doyle and Wang, 2019; Gurunathan et al., 2019). Among them, miRNA can affect the various stages of cerebral ischemia injury and can worsen or reduce brain damage through various ways.

**TABLE 1:** Exosomes for the treatment of other diseases.

Source	Component	Effect
BMSCs-Exos	miR-421	Treatment of retinal ischemic reperfusion injury (Mathew et al., 2019).
UCMSCs-Exos	—	Prevention of pyroptosis and repair of ischemic muscle injury through miR-421/circHIPK3/FOXO3a pathway (Yan et al., 2020).
UCMSCs-Exos	—	HIF1α enhanced fracture repair and angiogenesis in stable fracture rat model (Zhang et al., 2019).
MSCs-Exos	MiR-21-3p	Regulatory pathways related to clopidogrel liver injury (de Freitas et al., 2017).
MSCs-Exos	MiR- 181a	Inhibition of inflammatory response after myocardial I/R injury (Wei et al., 2019).
MSCs-Exos	MiR-221-3p MiR-294	Promoting repair of damaged heart (Khan et al., 2015; Coskunpinar et al., 2016; Sun et al., 2020).
MSCs-Exos	MiR-21	Down-regulation of PTEN expression and activation of PI3K/AKT signaling to reduce myocardial injury (Shi et al., 2018).
MSCs-Exos	MiR-21	Reprogramming Macrophages Post Myocardial Ischemia-Reperfusion Injury (Tan et al., 2021).
MSCs-Exos	MiR-30c-5p	Down-regulating BCL2-like 11 can improve myocardial injury, histopathological changes and apoptosis in rat I/R model (Meng et al., 2021).
MSCs-Exos	MiR-29a-3p	Upregulation of NRF2 and inhibition of CCNT2 attenuated ischemia reperfusion (Tian et al., 2021).
AMSCs-Exos	MiR-423-5p	Targeting Sufu mediates angiogenesis (Xu F. et al., 2019). Diagnosis and treatment of tumor (Shan et al., 2020; Jiang et al., 2021; Xue et al., 2022).

*MSCs, mesenchymal stem cells; BMSCs, bone mesenchymal stem cells; ADMSCs, adipose-derived mesenchymal stem cells; HUCMSC, human umbilical cord-derived mesenchymal stem cells; Exos, Exosomes; NRF2, Nuclear factor erythroid-2 related factor 2; CCNT2, Cyclin T2.*

### Exosome Treatment for Ischemic Stroke

**FIGURE 3:**
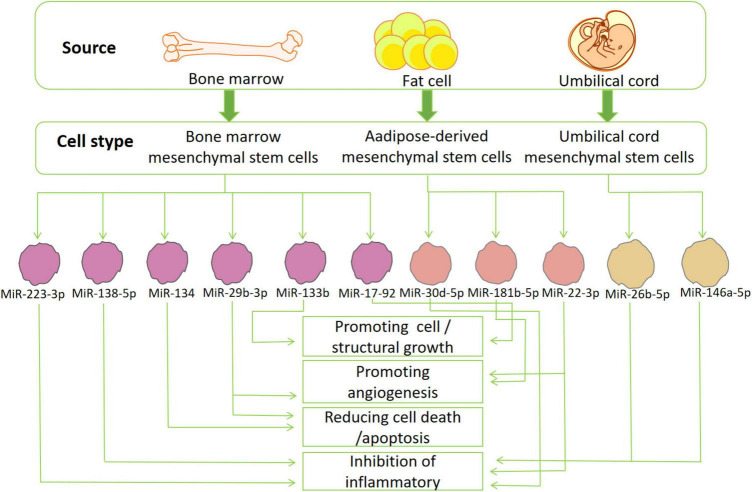
The source and function of stem cells: exosomes derived from various mesenchymal stem cells (bone marrow-, adipose-tissue and umbilical cord mesenchymal stem cells) secrete miRNA that can participate in many pathological or physiological processes of ischemic stroke. These miRNAs can reduce inflammation, oxidative stress, cell death/apoptosis, or promote the formation of blood vessels, cells and structures.

As mentioned before, the pathological response caused by IS is a complex process closely related to inflammation, calcium overload, and oxidative stress (Kalogeris et al., 2016; Enzmann et al., 2018; Castelli et al., 2021). This part of the article will elaborate on the different functions of miRNAs in different stroke-related pathological processes (Figure 3).

#### Decrease of Inflammation

During IS, reperfusion causes an inflammatory cascade, a key mechanism of secondary neuronal damage and death after the initial ischemic attack. As already discussed, changes in the brain tissue microenvironment affect the resident microglia transformation to M1 or M2 phenotypes (polarization) (Spellicy and Stice, 2021). Balancing the two polarizations of microglia is a promising method for the treatment of stroke. Experimental results reveal that BMSCs-exosomes (BMSCs-Exos) can reverse the CysLT2R-ERK1/2-mediated microglial M1 polarization, and thus inhibit microglial injury (Zhao et al., 2020b). Cysteinyl leukotrienes (CysLTs) are mainly produced by the decomposition of necrotic cells, acting as an effective inflammatory medium that aggravates the development of ischemic penumbra. BMSCs-Exos significantly inhibited the expression of CysLT2R in microglia after IS, reduced the secretion of pro-inflammatory cytokines, and increased the production of anti-inflammatory and neurotrophic factors (Zhao et al., 2020b). *In vitro* data suggest that miR-223-3p from BMSCs-Exos reduces inflammatory damage by inhibiting the polarization of microglia M1, and this is closely related to the down-regulation of the transcription and expression of cyyslt2r (Zhao et al., 2020a). MiR-223 is highly encapsulated in the exosomes released by MSCs and thus it is the most abundant miRNA in MSCs (Morales et al., 2022). Exosomal miR-223 can effectively promote the transformation of harmful M1 microglia induced by NMLTC4/ltd4 to beneficial M2 phenotypes, thereby increasing the expression of anti-inflammatory cytokines (Morales et al., 2022). Further experimental data suggest that miRNA-26b-5p derived from HUCMSCs exosomes target CH25H to inactivate TLR pathway, thereby inhibiting M1 polarization and at the same time attenuate autophagy-induced brain injury by promoting M2 microglia polarization (Jiang et al., 2018; Li G. et al., 2020). The results also revealed that the expression of miR-30d-5p could significantly inhibit the expression of Beclin-1 and Atg5- by targeting the 3′UTR levels of Beclin-1 and Atg5- and inhibit the ischemia-induced polarization of microglia to M1 (Jiang et al., 2018). All accumulating data suggest that the increase of the M2/M1 ratio in the brain has beneficial anti-inflammatory properties and neuroprotective effects (Gao et al., 2016; Kumar et al., 2016). In addition to changing the polarization of microglia in the brain, many other anti-inflammatory mechanisms have been discovered in recent years. Mechanism studies have shown that miR-146a-5p in exosomes derived from HUCMSCs alleviate neuroinflammation and neurological deficits caused by microglia after IS through IRAK1/TRAF6 (Zhang Z. et al., 2021). Overexpression of IRAK1 and TRAF6 is an important factor in the expression of pro-inflammatory genes and can activate the NFκB-a transcription factor. MiR-146a-5p down-regulates inflammation by binding to the 3′UTR of mRNA encoding the receptor proteins RAK1 and TRAF6. These (IRAK1 and TRAF6) are mainly involved in the toll-like receptor (TLR)-activated pathway, resulting in the expression of pro-inflammatory mediators (Wang et al., 2020). Exosomes-miR-542-3p also attenuates IS-induced apoptosis and ROS and inflammatory response by regulating TLR4 in glial cells (Cai et al., 2021). TLR4 is an important natural immune receptor, which can regulate the inflammatory response of multiple organs (Bruning et al., 2021). MiR-22-3p derived from exosomes of ADMSCs can inhibit inflammatory response and reduce cerebral ischemia injury by promoting macrophage M2 polarization and inhibit inflammation (Fang et al., 2021); inhibiting the activity of caspase-3, increasing the expression of Bcl-2 in neurons (Yu et al., 2015); reducing the expression of NF-κB co-activator NCOA1, and significantly inhibiting the activity of NF-κB (Yu et al., 2015); and reducing the apoptosis rate of cortical neurons (Yu et al., 2015). Exosomes from BMSCs with overexpression of miR-138-5p delay inflammation by inhibiting LCN2, an iron transporter involved in the response to brain injury (Deng et al., 2022). The miR-221-3p targeting ATF3 can significantly decrease the expression of pro-inflammatory cytokines (TNF-α, MCP-1, VCAM-1, IL-6) and chemokines (CCL2, CCL3) and block macrophage infiltration and microglia activation, thus potentially alleviating ischemia induced neuronal injury (Shan et al., 2021). The effects of the various exosome-derived miRNAs are summarized in Table 2.

**TABLE 2:** MiRNA regulates pathophysiology of ischemic stroke.

Effect	MiRNAs	Source	Pathway/factor
Inhibition of inflammatory	MiR-223-3p (Zhao et al., 2020a)	BMSCs	Down-regulation of CysLT2R transcription and expression.
	MiR-26b-5p (Li G. et al., 2020)	HUCMSCs	Inactivated TLR pathway targeting CH25H.
	MiR-30d-5p (Jiang et al., 2018)	ADMSCs	Targeting Beclin-1 and Atg5.
	MiR-146a-5p (Zhang Z. et al., 2021)	HUCMSCs	Targeting IRAK1/TRAF6 pathway.
	MiR-542-3p (Cai et al., 2021)	MSCs	Targeting TLR4.
	MiR-22-3p (Yu et al., 2015; Fang et al., 2021)	ADMSCs	Promote M2 polarization of macrophages. Inhibition of caspase-3 activity and Bax increased. Bcl-2 expression in neurons. Inhibition of NF-κB activity. Reducing the apoptosis rate of cortical neurons.
	MiR-138-5p (Deng et al., 2022)	BMSCs	Inhibition of LCN2.
	MiR-221-3p (Shan et al., 2021)	MSCs	Targeting ATF3.
Reducing oxidative stress	MiR-92b-3p (Xu L. et al., 2019)	MSCs	Reducing oxidative stress-induced neuronal damage.
Reducing cell death/apoptosis	MiR-29b-3p (Hou et al., 2020)	BMSCs	Down-regulation of Bax expression, cleavage of caspase3 and up-regulation of Bcl-2.
	MiR-26a-5p (Cheng et al., 2021)	MSCs	Targeting CDK6.
	MiR-134 (Xiao et al., 2018)	BMSCs	Negative regulation of caspase-8 dependent apoptosis pathway.
Promoting angiogenesis	MiR-29b-3p (Hou et al., 2020)	BMSCs	Down-regulation of PTEN. Activation of Akt signaling pathway.
	MiR-181b-5p (Yang et al., 2018)	ADMSCs	Up-regulates the protein expression of HIF-1α and VEGF. Downregulates the protein expression of TIMP3.
	MiR-210 (Lou et al., 2012; Meng et al., 2018)	MSCs	Targeting vascular endothelial growth factor signaling pathway.
Promoting cell/structural growth	MiR-133b (Lou et al., 2012)	BMSCs	Downregulation of CTGF.
	MiR-17-92 (Xin et al., 2017)	BMSCs	Activation of PI3K/Akt/mTOR/GSK-3β signaling pathway.
	MiR-22-3p (Zhang Y. et al., 2021)	ADMSCs	Inhibition of kdm6b-mediated BMP2/BMF axis.
	MiR-124 (Sun et al., 2013)	MSCs	Promoting differentiation of neural progenitor cells.

*MSCs, mesenchymal stem cells; BMSCs, bone mesenchymal stem cells; ADMSCs, adipose-derived mesenchymal stem cells; HUCMSC, human umbilical cord-derived mesenchymal stem cells.*

#### Reduction of Oxidative Stress

Brain cells will produce a large amount of ROS during ischemia and hypoxia. Oxidative stress is the sum of many pathological processes, such as mitochondrial damage, calcium overload and complement activation, which is an interactive cycle (Lin et al., 2016; Yin et al., 2016). Therefore, blocking one of the links could reduce neuronal death and neurological dysfunction caused by ischemia and hypoxia. MSCs can transfer its mitochondria to damaged endothelial cells, promote angiogenesis, reduce infarct volume, and improve functional recovery (Liu et al., 2019). In an *in vitro* model of cerebral ischemia, MSC-exosome-mediated-miR-92b-3p inhibited oxidative stress-induced neuronal damage, although the exact mechanism of action remains unclear (Xu L. et al., 2019). Recent studies have identified that receptor for advanced glycation end products (RAGE) is excessive in hypoxic cells of ischemic brain, exosomes can downregulate RAGE and miR-181a, and significantly reduce cerebral infarction area in an IS model (Kim et al., 2021). Meanwhile, recent studies have also reported that miR-451a can reduce ROS production by inhibiting the total expression of Rac1 and the formation of NOX (Li H. et al., 2021). Exosomes derived from human umbilical vein endothelial cells can reduce oxidative stress by inhibiting mitochondrial RNA-processing endoribonuclease in neurons through distance transfer of miR-206 and miR-1-3p (Zhong and Luo, 2021). However, studies on the potential of MSC-exosomes to block the generation of mitochondrial ROS alone are few. This may be a new research direction for the treatment of IS in the future.

#### Inhibition of Cell Death/Apoptosis

After IS, several brain cells die due to various pathological reactions. MiR-29b-3p in BMSCs-Exos inhibits cell apoptosis by reducing the expression of Bax, cutting caspase3 and up-regulating Bcl-2 (Hou et al., 2020), and promotes neuronal survival by promoting the anti-apoptotic signal cascades (Zhang Y. et al., 2021). MiR-26a-5p in MSC-exosomes inhibits microglial apoptosis by targeting CDK6 (Cheng et al., 2021). Similar to neurons, oligodendrocytes are highly sensitive to hypoxia, oxidative stress, inflammatory mediators, injuries, or injuries induced by infection. MiR-134 in the exosomes derived from BMSCs inhibits the apoptosis of oligodendrocytes by negative regulation of the caspase-8-dependent apoptosis pathway. Caspase8 and camp responsive element binding protein are specific targets of miR134 (Xiao et al., 2018). In addition to inhibiting the traditional apoptotic pathway, the latest research has revealed that circ_0000647 was elevated in the animal model of cerebral ischemia, and miR-126-5p was weakly expressed in cerebral cells under ischemia and hypoxia (Dai et al., 2022). Exosomes-circ_0000647 promotes apoptosis by targeting miR-126-5p to reduce TRAF3 (TRAF3 is the target gene of miR-126-5p) (Dai et al., 2022). The discovery of these new biomarkers may provide a new therapeutic target for IS in addition to contributing to a more detailed understanding of the pathogenesis of IS.

#### Stimulation of Angiogenesis

**FIGURE 4:**
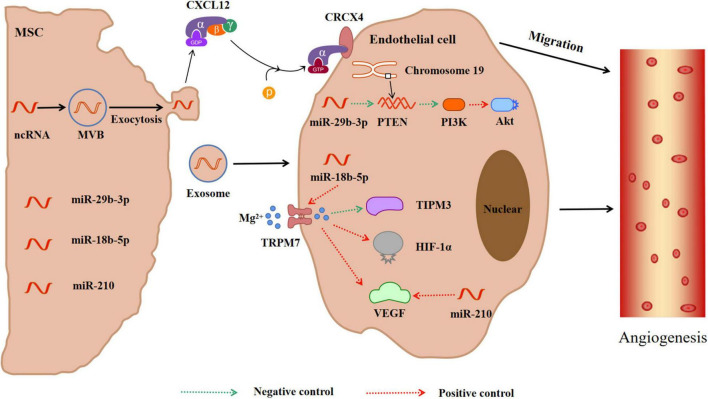
MiRNAs in exosomes secreted by mesenchymal stem cells promote angiogenesis in ischemic tissue. Meanwhile, CXCL12 secreted by exosomes can bind to CRCX4 and promote the migration of cerebrovascular endothelial cells to ischemic tissue. In addition, miRNAs in exosomes activate PTEN-PIK3-Akt or TRPM7-TIPM3/HIF-1α/VEGF pathways to promote angiogenesis. CXCL12 = SDF-1, Stromal cell derived factor 1; CRCX4, CXC receptor type 4; PTEN, Phosphatase and tensin homolog; PIK3, Phosphatidylinositol 3-kinase; Akt, Protein kinase B; TRPM7, Mg^2+^ transmembrane transport channel; TIPM3, Tissue inhibitor of metalloproteinase 3; HIF-1α, Hypoxia induce factor-1α; VEGF, Vascular endothelial growth factor.

The repair mechanism of damaged tissue requires effective EC regeneration and reinstatement of blood flow in damaged ischemic tissue. In order to improve the repair process, the EC in the innermost layer of the blood vessels will regenerate and proliferate to maintain blood supply in the tissues and tissue homeostasis (Baruah and Wary, 2019) and promote angiogenesis in ischemic areas (Figure 4). Data have revealed that within 21 days after ischemia, new blood vessels form at the edge of the ischemic area and gradually extend to the ischemic center through budding (Li X. et al., 2020). Consequently, enhancing vascular proliferation in ischemic brain tissue has potential therapeutic prospects. Proteomics analysis indicated that exosomes released from MSCs have angiogenic paracrine effects (Ghafouri-Fard et al., 2020). The collateral circulation can protect ischemic brain tissue by increasing the blood supply of ischemic penumbra (Chen W. et al., 2022). Exosomes-CXC receptor type 4 can enhance the regeneration and tube-like structure formation of microvessel endothelial cells (MECs) (Li X. et al., 2020). CXC motif CR4 belongs to the G protein-coupled receptor superfamily and participates in homing of many cells (Shichinohe et al., 2007). CXCL12 promotes angiogenesis *in vivo*. CXCL12 can bind to CXCR4 on cells expressing CXCR4, including brain MECs. This promotes the migration of brain MECs to ischemic tissues and improves the repair of brain tissue (Liu Q. W. et al., 2022). MiR-29b-3p in exosomes derived from BMSC targets PTEN and activates Akt-signaling pathway to accelerate angiogenesis in IS (Hou et al., 2020). The gene for PTEN, located on human chromosome 19, encodes a double phosphatase that dephosphorylates protein and lipid substrates (Garcia-Junco-Clemente and Golshani, 2014). Moreover, PTEN negatively regulates the phosphatidylinositol 3-kinase/protein kinase B (PI3K/Akt) signaling pathway, thereby accelerating angiogenesis after IS (Qu et al., 2016). MiR-29b-3p negatively regulates PTEN, activates Akt-signaling pathway, and accelerates angiogenesis (Hou et al., 2020). ADMSCs-exosomes (AMSCs-Exos) promote the angiogenesis of BMSCs after IS through the miR-181b-5p/TRPM7 axis. The Mg^2+^ transmembrane transport channel (TRPM7) is the direct target of miR-181b-5p. MiR-181b-5p up-regulates the expression of HIF-1α and VEGF by targeting TRPM7, and down-regulates the expression of TIMP3 (Yang et al., 2018). Moreover, miR-210 can also promote microvascular angiogenesis, which is consistent with the up-regulation of the vascular endothelial growth factor (VEGF) protein. The miR-210 is the main miRNA induced by hypoxia, which promotes angiogenesis mediated by the VEGF signaling pathway (Lou et al., 2012; Meng et al., 2018).

#### Stimulation of Cell and Structure Growth

**FIGURE 5:**
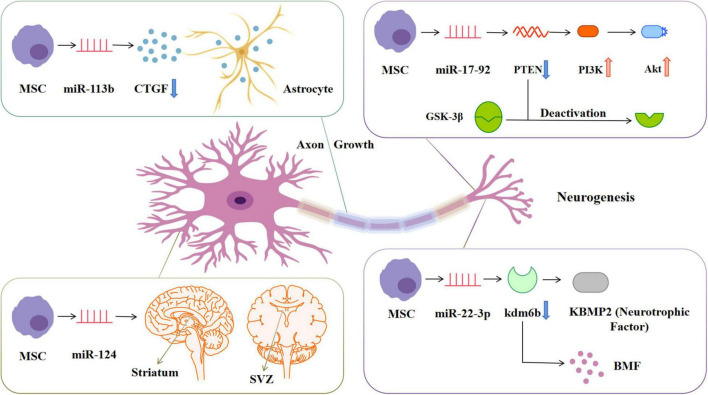
MiRNAs in exosomes secreted by mesenchymal stem cells stimulate cell and structure growth. miRNA-133b can down-regulate the expression of CTGF in astrocytes, reduce the formation of glial scar and promote the remodeling of myelin sheath. miRNA-17-92 can down-regulate the expression of PTEN, thereby activating the PIK3-Akt pathway and inactivating GSK-3β, promoting the growth of neuronal axons. miRNA-124 promotes neurogenesis in SVZ and striatum regions. miRNA-22-3p can inhibit KDM6B-mediated KBMP2/BMP pathway and play a neurotrophic role. CTGF, Connective tissue growth factor; PTEN, Phosphatase and tensin homolog; PIK3, Phosphatidylinositol 3-kinase; Akt, Protein kinase B; GSK-3β, Glucogen synthase kinase-3β; SVZ, Subventricular zone; KDM6B, Lysine(K)-specific demethylase 6B; KBMP2, Neurotrophic factor; BMP, Bone morphogenetic protein.

In the injured central nervous system, glial scars are the main obstacle to axon regeneration, and they consist of extracellular matrix deposition and proliferation of reactive astrocytes (Figure 5). The latter significantly express connective tissue growth factor, which is down-regulated by miR-133 (Xin et al., 2013). The overexpression of miR-133b in exosomes secreted by BMSCs can stimulate the secondary secretion of exosomes from astrocytes and reduce the thickness of glial scars, thus promoting the recovery of neurological function and increasing neural plasticity (Vizoso et al., 2017). MiR-17-92 from BMSCs-Exos enhances axonal myelin remodeling and motor electrophysiological recovery after stroke by activating the PI3K/Akt/mTOR/GSK-3β signaling pathway. The overexpression of miR-17-92 promotes the axon growth of primary cortical neurons by down-regulating PTEN and subsequently activating the PI3K/Akt/mTOR signaling pathway (Xin et al., 2017). MiR-17-92 promotes oligodendrogenesis during neurodevelopment and enhances stroke-induced neurogenesis (Xin et al., 2021). Axonal regeneration after spinal cord injury can also be regulated by the PTEN/PI3K/mTOR signaling pathway (Hu et al., 2022). GSK-3β plays a key role in axon regeneration. Inactivation of GSK-3β promotes axonal growth and neurological recovery in the central nervous system (Arciniegas Ruiz and Eldar-Finkelman, 2021; Rodriguez-Jimenez et al., 2021). MiR-22-3p derived from adipose-tissue MSC exosomes can alleviate cerebral ischemia injury by inhibiting the BMP2/BMF axis mediated by kdm6b. KBMP2 is a neurotrophic factor that can induce the growth of dopaminergic neurons in the midbrain *in vivo* and *in vitro* and play a neurotrophic role (Zhang Y. et al., 2021).

After cerebral ischemia, the expression of miR-124 in the ischemic penumbra increases (Yang et al., 2017). MiR-124 is the most abundant miRNA in the brain and a determinant of the fate of subventricular zone (SVZ) neurons. It mediates stroke-induced neurogenesis in adult SVZ and striatum. The ectopic expression of miR-124 in the cortex promotes the differentiation of neural progenitor cells into neuronal lines, and further improves the anti-ischemic ability through cortical neurogenesis (Zhou and Qi, 2021). Recent studies have reported that CircOGDH (cyclic RNA derived from ketoglutarate dehydrogenase) is highly expressed in plasma exosomes of patients with IS, and CircOGDH interacts with miR-5112 in primary cortical neurons to increase neuronal damage (Liu Y. et al., 2022). By contrast, knockdown of CircOGDH significantly enhanced neuronal viability under ischemic conditions. Therefore, CircOGDH and miR-5112 may be potential therapeutic targets for regulating the viability of ischemic neurons (Liu Y. et al., 2022).

In summary, MSC-exosomes are mostly derived from BMSCs and ADMSCs, while HUCMSCs are used less frequently. However, the experimental results revealed that HUCMSCs were easier to obtain than BMSCs, demonstrated higher viability, were more easily accepted by patients, and were less susceptible to immune-mediated graft rejection (Shang et al., 2021). Additionally, although HUCMSCs are considered more primitive than BMSCs, they have not demonstrated teratoma induction, although they demonstrated immunoregulatory ability. In the clinical research setting, HUCMSCs have the advantage of preventing pain during bone marrow extraction, thus improving volunteer compliance (Eiro et al., 2021). The increasing number of umbilical cord blood preserved in new-borns also suggests an increased possibility for future clinical implementation of this method.

## Novel Application of Stem Cells in Clinical Treatment

MSCs have paracrine and immune regulation functions, which can change the microenvironment of damaged brain tissue and transforms it to a more regenerative and less inflammatory environment. MSCs can promote cell migration, angiogenesis, immune regulation, nerve protection, and nerve circuit reconstruction (Law et al., 2021). Moreover, many preclinical research evidences have demonstrated that MSCs promote the clinical transformation of MSCs in the treatment of IS. Recent randomized controlled trials (RCTs) and meta-analysis have suggested that stem cell-based therapy can improve neurological deficits and activities of daily living in patients with IS, although its benefits are still limited (Li Z. et al., 2020). Moreover, intravenous injection of MSCs has been proven to be safe, well tolerated, and relatively feasible. However, several common limitations exist for current RCTs, such as small sample size (Law et al., 2021), long-term waiting for MSC culture, delayed assessment of the treatment group, age of participants, randomization time, heterogeneity of ischemic brain injury site, and severity (Chung et al., 2021). Therefore, further randomized, double-blind, large-scale clinical trials are necessary to evaluate the long-term efficacy and safety of this treatment method.

## Conclusion and Perspective

IS is the leading cause of global morbidity and mortality. Exosomes act as natural carriers of biologically active molecules such as bioactive molecules, various proteins, and coding and non-coding RNAs and can transmit information between cells and tissues while lacking immunogenicity (Zhou et al., 2022). Exosome-MiRNA regulates transcriptional and post-transcriptional gene expression and modulates various tissue repair, inflammation, hypoxia, and angiogenesis pathways (Lee et al., 2021). The most important effect is on the tissue microenvironment by mediating various physiological/pathological pathways. A typical example is the repair of ischemic brain injury, which is the focus of this paper. We briefly introduced the potential mechanisms of MSC-exosome-miRNA-mediated nerve function recovery, such as anti-apoptosis, anti-inflammation, nerve plasticity, and angiogenesis. However, the research of MSCs and exosomes in the treatment of IS is still in its infancy in clinical practice. Future clinical trials should be conducted to answer how several MSC-exosomes are obtained, how the survival of most transplanted cells can be increased, how the infarction of most MSC-exosomes can be guided, and how the distribution of MSC-exosomes without any tissue damage is detected (Jiang et al., 2022). Additionally, almost all experiments were conducted in healthy animals. Some highly related complications, such as diabetes, hypertension, hyperlipidemia, and heart disease, may need to be considered because they will affect the formation and treatment of stroke (Li J. Y. et al., 2021). Finally, the extension of exosome half-life and the improvement of the targeting ability should be considered in its application in medicine. At the same time, overcoming drug resistance and expanding the clinical application of conventional drugs are also crucial (Yang et al., 2021). Therefore, stem cells and exosomes may be a promising new treatment in clinical practice, which needs further research.

## Author Contributions

XH queried the research background. ZP and JS participated in the understanding of exosome development. WH carried out literature reading on the clinical manifestations of ischemic stroke and the advantages and disadvantages of existing treatment. RG and LS carried out language grammar and logic examination of manuscripts. FZ and PS participated in the review of medical professional knowledge of manuscripts. LS and YX conceived the study, participated in its design and coordination and helped draft the manuscript. All authors read and approved the final manuscript.

## Conflict of Interest

The authors declare that the research was conducted in the absence of any commercial or financial relationships that could be construed as a potential conflict of interest.

## Publisher’s Note

All claims expressed in this article are solely those of the authors and do not necessarily represent those of their affiliated organizations, or those of the publisher, the editors and the reviewers. Any product that may be evaluated in this article, or claim that may be made by its manufacturer, is not guaranteed or endorsed by the publisher.

## Abbreviations

AMSCs: adipose-derived mesenchymal stem cells
Arg-1: arginase-1
Atg5: autophagy-related gene 5
Bax: bcl-associated X protein
BBB: blood brain barrier
Beclin-1: autophagy gene beclin 1
BMF: bone marrowfacror
BMP2: bone morphogenic protein 2
BMSCs: bone marrow-derived mesenchymal stem cells
CCL2/CCL3: chemotactic factor
CH25H: cholesterol-25-hydroxylase
CINC: cytokine-induced neutrophil chemoattractant
CircHIPK3: circular RNA HIPK3
CXCL1/CXCL12: chemotactic factor
CXCR4: CXC motif chemokine receptor type 4
CysLT2R: cysteinyl leukotriene receptor 2
CysLTs: cysteinyl leukotrienes
EC: endothelial cell
ERK1/2: extracellular signal-regulated kinase 1/2
Exos: exosomes
Foxo3a: forkhead box o3a
GRO: growth-related oncogene
GRO-α: growth-related oncogene α
GSK-3β: glucogen synthase kinase-3 β
HIF-1α: hypoxia induce factor-1 α
HUCMSCs: human umbilical cord-derived mesenchymal stem cells
I/R: ischemia and reperfusion
IL-1: interleukins-1
IL-10: interleukins-10
IL-1β: interleukins-1 β
IL-4: interleukins-4
IL-6: interleukins-6
INF-γ: interferon-y
IP: ischemic penumbra
IRAK1: interleukin receptor associated kinase 1
IS: ischemic stroke
KC: keratinocyte-derived chemokine
LTC4/LTD4: leukotriene C4/D4
MCP1: mononuclear chemotaxis protein-1
MSC-EV: mesenchymal stem cells-external vesicles
MSCs: mesenchymal stem cells
mTOR: mammalian target of rapamycin
NFκB-a: nuclear factor kappa B-a
NOS: nitric oxide synthase
NOX: reduced nicotinamide adenine dinucleotide phosphate oxidase
PETN: phosphatase and tensin homolog
PI3K: phosphatidylinositol 3-kinase
PKB/AKT: protein kinase B
PTEN: phosphatase and tensin homolog
ROS: reactive oxygen species
rt-PA: recombinant tissue plasminogen activator
STAT6: signal transducers and activators of transcription 6
SVZ: subventricular zone
TIMP3: tissue inhibitor of metalloproteinase 3
TLR: toll-like receptor
TLR4: toll-like receptor 4
TNFα: tumor necrosis factor α
TRAF6: tumor necrosis factor receptor-associated factor 6
TRPM7: Mg^2+^ transmembrane transport channel
VCAM-1: vascular cell adhesion molecule-1
VEGF: vascular endothelial growth factor.
